# Phenology Information Contributes to Reduce Temporal Basis Risk in Agricultural Weather Index Insurance

**DOI:** 10.1038/s41598-017-18656-5

**Published:** 2018-01-08

**Authors:** Tobias Dalhaus, Oliver Musshoff, Robert Finger

**Affiliations:** 1 Agricultural Economics and Policy Group, ETH Zürich, Sonneggstrasse 33, 8092 Zürich, Switzerland; 2 Department of Agricultural Economics and Rural Development, Georg August University Göttingen, Platz der Göttinger Sieben 5, 37073 Göttingen, Germany

## Abstract

Weather risks are an essential and increasingly important driver of agricultural income volatility. Agricultural insurances contribute to support farmers to cope with these risks. Among these insurances, weather index insurances (WII) are an innovative tool to cope with climatic risks in agriculture. Using WII, farmers receive an indemnification not based on actual yield reductions but are compensated based on a measured weather index, such as rainfall at a nearby weather station. The discrepancy between experienced losses and actual indemnification, basis risk, is a key challenge. In particular, specifications of WII used so far do not capture critical plant growth phases adequately. Here, we contribute to reduce basis risk by proposing novel procedures how occurrence dates and shifts of growth phases over time and space can be considered and test for their risk reducing potential. Our empirical example addresses drought risks in the critical growth phase around the anthesis stage in winter wheat production in Germany. We find spatially explicit, public and open databases of phenology reports to contribute to reduce basis risk and thus improve the attractiveness of WII. In contrast, we find growth stage modelling based on growing degree days (thermal time) not to result in significant improvements.

## Introduction

Agricultural insurance solutions are an important tool for farmers to manage risks. Among a wide set of available insurance solutions^1^ weather index insurances (WII) have recently emerged as promising alternative to classical damage based insurance solutions. For WII the payout made to the farmer is based on a measured index, e.g. precipitation at a weather station, and is not directly based on yield or income losses experienced by the farmer. Thus, WII overcome asymmetric information problems of classical insurance schemes, because farmer and insurance company have equal information about the weather risk and are unable to manipulate the insured value (the weather)^2^. In addition, compensation of farmers only requires information of weather records and is thus fast and cheap. Hence, WII have a large potential in both developed and developing countries and can contribute to better farm-level risk management and more efficient use of natural resources^3,4^. However, index insurances not necessarily lead to accurate compensation of yield losses and thus might fail to payout if farmers experience income losses. This phenomenon is denoted as basis risk and constitutes a significant adoption hurdle of these products by farmers. Basis risk can be separated into three components: (1) Geographical/spatial basis risk occurs if index is measured with spatial distance to production location^5^. (2) Design basis risk is a result of taking an index that is an inadequate predictor of yield losses^6^. (3) Temporal basis risk captures the imperfect choice of the time frame for index measurement^7,8^. In this paper, we suggest novel approaches to reduce temporal basis risk.

Temporal basis risk mainly occurs because WII does not reflect the actual growth stage that is sensitive to specific weather, e.g. droughts. The measurement period for the weather index has to be specified in the insurance contract by both parties before the growth period of the crop starts. As the most straightforward procedure, periods over which the index is measured are thus often chosen to reflect particular calendar periods (e.g. specific weeks or months). These fixed time windows can only roughly approximate crop specific growth phases^9^. Moreover, the occurrence dates of growth phases are not constant across time and space, because weather conditions can cause large shifts in the actual occurrence of these periods^10,11^. The resulting misspecification of insurance periods results in biased WII payout determination and therefore weak risk reducing properties hampering insurance uptake across risk averse farmers^12^. So far, only few studies have suggested approaches aiming to reduce temporal basis risk of WII, following more flexible index designs, i.e. by considering shifts of crop growth phases over time and space^13,14^. In this respect, ‘flexible’ implies to implement yearly changing insurance periods according to the actual occurrence dates of vulnerable growth phases^10^. First, Kapphan *et al*.^13^ used growing degree days (GDD) to model occurrence dates of emergence, vegetative period, grain filling and maturity in corn production based on thermal time. They evaluated the performance of their WII based on simulated corn yield and weather scenarios. Second, Conradt *et al*.^14^ used GDD to simulate occurrence dates of tillering, shooting and ear emergence in spring wheat. They tested the risk reducing properties of the resulting WII based on a case study in Kazakhstan. Both approaches allow for fine scaled estimates of multiple growth phases. Furthermore, Dalhaus and Finger^15^ suggest to use observations from a phenological network of farmland in the farms’ region to find winter wheat’s occurrence dates of stem elongation, ear emergence and milk ripeness. They tested their WII based on a case study in central Germany. The latter approach accounts additionally for a maximum of comprehensibility for the farmer, which is considered as key success factor in WII^16,17^ So far, no study has compared different approaches to consider crop growth phases in WII design.

We here use an empirical example of a WII against drought risk in winter wheat production in Germany to compare existing and propose new approaches to reduce temporal basis risk. In winter wheat production, especially phases of low water supply during “reproductive and grain-filling” limit the development of the plant^18^. Farooq *et al*.^18^ review outcomes of several contributions concerning yield reduction due to drought taking place at different developmental stages in winter wheat. Their findings indicate, that wheat is most vulnerable to drought during the phase from ‘stem elongation’ to ‘anthesis’^18,19^. Within this phase, assimilates are to a large extent used to develop grains^20^. Hence, drought induced leaf senescence^21,22^, reduced carbon uptake due to stomata closure^23^ as well as shortening of grain filling period^20^ decrease grain number and grain weight, thus reducing final yield outcome.

In this study, we aim to test and compare different approaches to find the occurrence dates of these phases and use this information to reduce temporal basis risk of WII. We focus on the following crop growth stage modeling and different phenology observation networks (see also Table 1 for comparative features).

- **Growing Degree Days:** Plants are expected to require a plant and growth stage specific temperature load to reach a certain growth stage. The growing degree days approach helps to model this based on observed temperature data. Using the GDD model we are able to estimate the occurrence dates of the drought sensitive period between stem elongation and anthesis of winter wheat.
- **Yearly Phenology Reporters:** Publicly provided open dataset of plant growth stage occurrence dates. The network comes with a high spatial density and a detailed reporting procedure including various different growth stages. Data is published at the end of the calendar year (denoted as ‘Yearly Reporter’ henceforward). Using yearly reporters’ data we are able to derive region specific information on the actual occurrence dates of the drought sensitive growth stages stem elongation and ear emergence in winter wheat.
- **Immediate Phenology Reporters:** Publicly provided open dataset of plant growth stage occurrence dates. The network however comes with a lower spatial density and less reported growth phases compared to the latter network. Data is published directly after observation (For a detailed explanation of all three approaches, see section ‘Determination of water sensitive growth stages’.) (denoted as ‘Immediate Reporter’ henceforward). Using immediate reporters’ data we are able to derive region specific information on the actual occurrence dates of the drought sensitive growth stages stem elongation and ear emergence in winter wheat.

**Table 1:** Characteristics of the different Approaches to account for Drought sensitive Growth Stages in WII Design.

GDD	Yearly Reporter	Immediate Reporter
Simulation	Observation	Observation
356 Weather Stations	1,200 reporters	400 Reporters
Numerous growth phases	7 growth phases for winter wheat	6 growth phases for winter wheat
Simulation of Anthesis occurrence possible	Anthesis not reported	Anthesis not reported
Immediate calculation	Available at the end of the year	Available immediately
Vernalization^a^ not considered	See Fig. 2 for the spatial distribution in the case study region	See Fig. 3 for the spatial distribution in the case study region

^a^Coolness requirement of winter crops to induce generative growth phases. GDD Approach does not distinguish between winter and spring temperature loads^29,30^.

To this end, we add to the current discussion of utilizing (big) data sources to support more efficient insurance solutions and thus sustainable agriculture^24,25^.

More specifically we aim to answer the following research questions

- **RQ1:** Which approach to explicitly consider yearly changing insurance periods reduces farmers’ financial exposure to drought risk compared to a ‘no insurance’ scenario?
- **RQ2**: Which approach to explicitly consider yearly changing insurance periods fits best to reduce temporal basis risk of weather index insurance?

We use expected utilities (EU) of insured farmers as risk measure to test for a reduction in the financial exposure to drought risk. We conduct this assessment for different scenarios of farmers’ level of risk aversion. Our approach is particularly focused on the relevance of WII to reduce downside risks, i.e. the compensation of extreme yield losses, by utilizing power utility function to calculate expected utilities and the use of quantile regressions to obtain critical parameters of the WII such as tick size^6^. Our empirical example is based on farm-level wheat yield data for northern Germany, with a focus on drought risks. We conclude with a critical discussion on the applicability of the various approaches considered here, with respect to the insured crop, data availability and potential further research paths.

## Results

For summary statistics on differences between the three approaches and WII contracts see the respective section of the online supplementary file.

We test for statistical significance of i) the ability of WII solutions to reduce farmers’ financial exposure to drought risk compared to no insurance (RQ1) and ii) differences across the different WII specifications used here (RQ2). Table 2 shows Wilcox test results of the risk reducing properties of the different insurance products compared to the uninsured case. This assessment is based on average values of expected utilities across all considered farms and a fair insurance premium. We find that both WII based on phenology reporting data highly significantly increased farmers’ expected utility and thus reduce the financial exposure to drought risk. This result holds over all implemented levels of risk aversion. Note that for risk neutrality (risk aversion being equal to zero) no improvement can be obtained from any insurance with fair insurance premiums. Regarding WII based on GDD estimated growth stages we could not detect any significant changes in expected utility compared to the ‘no insurance’ base scenario. Hence, GDD based WII did not reduce the financial exposure to drought risk in our empirical example of winter wheat production in Germany.

**Table 2:** Results RQ1: Tests for Risk reducing Properties of different WII compared to ‘no insurance’ Reference Scenario.

Coefficient of relative risk aversion α	Yearly Reporter	Immediate Reporter	GDD…
	H_0_:EU_year_ ≥ EU_noins_	H_0_:EU_imm_ ≥ EU_noins_	H_0_:EU_GDD_ ≥ EU_noins_
	p- value		
0 (risk neutral)	0.62	0.29	0.93
0.5	3.48·10^−2^	6.31·10^−2^	0.84
1	2.51·10^−2^	7.49·10^−3^	0.64
2	6.73·10^−3^	9.93·10^−3^	0.60
3	5.72·10^−3^	1.42·10^−2^	0.51
4 (extremely risk averse)	5.28·10^−3^	1.31·10^−2^	0.53

EU_year_: Vector of expected utility values of insured farmers using yearly phenology reporters’ data.

EU_imm_: Vector of expected utility values of insured farmers using immediate phenology reporters’ data.

EU_GDD_: Vector of expected utility values of insured farmers using growing degree days modeling.

EU_noins_: Vector of expected utility values of uninsured farmers.

Table 3 displays the results of comparisons between the different approaches. We find no difference in the risk reducing properties between different phenology reports. This result reveals that the benefits of using phenology reports in WII are independent of the reporting schemes. Compared to WII based on GDD approach, both phenology reporter based WII performed significantly better and thus reduced temporal basis risk.

**Table 3:** Results RQ2: Comparing Risk reducing Properties between WII.

Coefficient of relative risk aversion α	H_0_:EU_year_ ≥ EU_imm_	H_0_:EU_year_ ≥ EU_GDD_	H_0_:EU_imm_ ≥ EU_GDD_
	p-value		
0 (risk neutral)	0.50	0.23	0.17
0.5	0.27	3.77·10^−2^	5.97·10^−2^
1	0.35	3.77·10^−2^	3.94·10^−2^
2	0.13	2.38·10^−2^	4.73·10^−2^
3	0.10	1.98·10^−2^	5.97·10^−2^
4 (extremely risk averse)	0.10	1.98·10^−2^	8.29·10^−2^

EU_year_: Vector of expected utility values of insured farmers using yearly phenology reporters’ data.

EU_imm_: Vector of expected utility values of insured farmers using immediate phenology reporters’ data.

EU_GDD_: Vector of expected utility values of insured farmers using growing degree days modeling.

EU_noins_: Vector of expected utility values of uninsured farmers.

All results presented here show the differences between the three WIIs with respect to their ability to reduce temporal basis risk and thus increase farmers’ expected utility. Within our online supplementary file we present results on the magnitude of the here identified effects (see Table A4 of the online supplementary file).

## Discussion

This study is the first comparing different WIIs that explicitly consider managing drought risk in single stages of plant growth. In our approach, insurance periods vary across time and space according to the occurrence dates of the growth stages stem elongation, anthesis and ear emergence. Our results reveal improvements for WII schemes by reducing temporal basis risk. Drought risks are expected to become more pronounced for arable farmers in Europe in the future. Thus, developing functioning WII insurance solutions is considered as viable climate change adaptation tool^26,27^.

Using phenological observations to suit WII to agronomical plant development highly significantly decreased farmers’ risk exposure and thus increased farmers’ expected utility compared to both, using GDD based WII and to the ‘no insurance’ reference scenario. However, both phenology reporting networks did only provide information about the growth stage of ‘ear emergence’ and not about the highly drought sensitive growth stage of ‘anthesis’, which can be estimated by the GDD approach^28^. More specifically, GDD based approaches allow a substantially finer assessment of crop growth stages than phenology observations. Nevertheless, the GDD approach failed to properly estimate the occurrence of ‘anthesis’ and risk reducing properties of the reporters based WII remained strong. The fact that both reporting networks, which have different reporting procedures and network densities, showed a relatively similar performance, underlines the robustness of our results to changes in the reporting procedure and station density. With respect to timely insurance payouts in the case of loss events, we would like to clearly emphasize that immediate reporters, which publish their findings right after occurrence, constitute the preferable option compared to yearly reporters. Timely compensation of losses to avoid illiquidity is considered as key requirement of crop insurances to avoid illiquidity. However, including the growth stage of ‘anthesis’ in the phenology reporting system would potentially further increase the risk reducing properties.

Concerning the usage of GDD to find appropriate WII periods, we found several drawbacks that have to be considered. Thus, GDD estimate of the occurrence dates of ‘stem elongation’ was considerably too early. As a result, rainfall in the insured period was considerably higher due to a longer insurance period and a shift into a more wet time of the year. Hence, drought risk was underestimated and farmers received fewer payouts resulting in low risk reducing properties (Tables 1 and 2, A2 and A3). GDD models might be improved using expert knowledge or additional experimental data. Furthermore, crop modelling approaches can provide valuable information to derive estimates for the occurrence dates of plant growth stages considering differences in the impact of winter and spring temperature loads (vernalization) and length of the day (photoperiodism)^29–32^. Thus, methods to reduce temporal basis risk must be selected crop specific, based on their ability to find occurrence dates of growth stages for the specific crop. Yet, these possible advances have to be aligned to findings that more complex WII solutions lead to lower acceptance on farmers’ side^16,17^.

Public institutions surveying phenological development of plants, i.e. the occurrence dates of growth stages, exist in many regions that are important crop insurance markets (see van Vliet *et al*.^33^, for Europe or Morellato *et al*.^34^ for South and Central America). However, despite their availability and the fact that all approaches tested could be easily implemented in current practical index insurance schemes, none of them has been considered in practice so far. This reveals a massive potential for improvements especially for WII products. This is particularly valid for countries such as the USA, where the market for WII is well established (premiums paid for WII exceeded 284 m USD in 2016, (www.rma.usda.gov) and various data sources on crop phenology are not yet used in WII (www.usapn.org).

WII currently are tested in many developing countries where the availability of phenology information might be limited and where the impacts of drought might be more severe^35,36^. Here, crop specific methods to find the occurrence dates of sensitive growth stages might be implemented. Whereas, in our case study the availability of cheap real-time phenology observations constitutes the most cost-effective and from farmer’s perspective comprehensible tool, it might be worth using more complex approaches in case of phenology data scarcity. In this respect also alternatives to GDD approach such as ‘biometeorological time’ or ‘physiological days’ as suggested by Saiyed *et al*.^37^ or satellite imagery^38^ could further reduce temporal basis risk. Improving WII by integrating crop modelling seems key in developing better insurance solutions for countries where phenology data is scarce. Moreover, validating GDD models using regional phenological observations could be a practical way to bring together advantages of both approaches. Consequently, our study discloses a variety of ways to include temporally flexible index designs.

Moreover, our findings contribute to the ongoing debate on the inclusion of novel (big) data sources in agricultural decision making in general and agricultural insurance in particular^24^. Within the broader picture of smart farming, where “aspects of technology, diversity of crop and livestock systems, and networking and institutions […] are considered jointly”^25^, we contribute a practical application that combines large and open datasets, crop modelling and meteorological applications with agronomical knowledge. Our findings are thus expected to stimulate further research but also business opportunities in the field of agricultural risk assessment and risk management.

Finally, our findings contribute to improve risk management options based on WII. But, individual risk management options should be compared and embedded in a whole farm analysis. For estimating the optimal risk management strategy coping with various perils a more holistic framework might be applied, taking into account the whole crop rotation, livestock production, the financial situation as well as off farm income, i.e. whole farm/ household income^39^.

## Methods and Data

### Design of the Weather Index Insurance

We aim to develop a WII that reduces the exposure to drought risk which frequently affects winter wheat yields in our study region^40^ (see section “Farm Level Yield Data” for a description of the underlying dataset). Thus, winter wheat yield *y* is displayed as a function of weather index *r*, in our case the sum of precipitation within a drought sensitive growth stage:

1 $$y=g(r)+\varepsilon $$

More specifically, we implement a cumulative precipitation index ${r}_{tik}^{R}$, which represents the sum of precipitation within a specific period^41,42^:

2 $${r}_{tik}^{R}=\sum _{d=start}^{end}{R}_{ti}^{d}$$

${r}_{tik}^{R}$ denotes the precipitation index of farm *i* in year *t* and insurance product *k* ∈ [GDD, Yearly Reporter, Immediate Reporter], summing up daily rainfall ${R}_{ti}^{d}$. Further, d = d_start_ and d = d_end_ mark start and end-dates of accumulation period and should be tailored to water sensitive growth stages. We especially aim to improve flexible start and end date detection by testing three different approaches to find these dates based on both phenological observation networks and crop growth stage modeling. By specifically suiting the weather index in equation 2 to the drought sensitive growth stages, we avoid to include damaging effects of excessive rainfall, which can also be reflected by a rainfall sum index^11,43^.

Using a European put option design WII is suited to indemnify losses caused by low precipitation events. European options are financial products that give the owner the right of exercising the option at a specific point in time. The owner then receives a payout depending on a payout function. In the case of a put option, the insurance payout begins if a specific strike level ${S}_{ik}$ of precipitation is undercut and rises depending on the options’ ticksize ${T}_{ik}$ (payout per missing index value, in our case mm precipitation). The insurance payout ${\pi }_{tik}^{put}\,$is determined by ${\pi }_{tik}^{put}=P\cdot [{T}_{ik}\cdot \,\max \{({S}_{ik}-{r}_{tik}^{R}),0\}]$, where *P* denotes the winter wheat price (Note that we assumed the winter wheat price to be 15.80 €/dt (dt denotes deciton, i.e. 100 kg)^44^, our results are robust against changes in *P* as shown in Tables A5–A8 of the online supplementary file)

Extending equation 1, wheat yield ${y}_{ti}$ of farm *i* is assumed to be random and stochastically dependent on weather index ${r}_{tik}^{R}\,\,$and an error term ${\tilde{\varepsilon }}_{ti}$:

3 $${y}_{ti}={c}_{ik}+{\beta }_{ik}\cdot {r}_{tik}^{R}+{\tilde{\varepsilon }}_{tik}$$

${c}_{i}$ is a constant intercept and ${\beta }_{i}$ the slope coefficient of the rainfall index variable that can be interpreted as the influence of rainfall index ${r}_{tik}^{R}$ on yields ${y}_{ti}$, both randomly distributed across the years.

We define strike level ${S}_{i}$ as the estimated rainfall value related to the farm individual mean yield $\bar{y}$ $({S}_{ik}={{g}_{ik}}^{-1}({\bar{y}}_{ik}))$. More specifically we insert coefficient estimates ${\hat{\beta }}_{ik}$ (which represents options’ ticksize *T*_*ik*_) and $\widehat{{c}_{i}}$ together with the mean yield ${\bar{y}}_{i}$ into equation 3 and solve for the corresponding rainfall index value ${r}_{ik}^{R}$ that marks the strike level of rainfall ${S}_{ik}$.

Both strike level and ticksize are obtained from quantile regression (QR) outcome, recently suggested by Conradt *et al*.^9^, estimated for each farm separately. The estimation problem is defined as:

4 $${\hat{\beta }}_{ik}(\tau )=\text{arg}\mathop{\min }\limits_{{\beta }_{ik}\in {\mathbb{R}}}(\tau \cdot \sum _{{y}_{i}\ge {\beta }_{ik}\cdot {r}_{ik}^{R}}|{y}_{i}-{\beta }_{ik}\cdot {r}_{ik}^{R}|+(1-\tau )\cdot \sum _{{y}_{i} < {\beta }_{ik}\cdot {r}_{ik}^{R}}|{y}_{i}-{\beta }_{ik}\cdot {r}_{ik}^{R}|)$$

QR focuses on a quantile of interest defined by $\tau$ and is comparably robust to outlier values as it minimizes the absolute distance between fitted values and residuals. We follow Conradt *et al*.^6^ and chose $\tau$ = 0.3 and specially suit the regression on low yield outcomes. We use the statistical software environment R-statistics^45^ with the additional package ‘quantreg’^46^. For a detailed description of using quantile regression in weather index insurance design see Conradt *et al*.^6^ and Dalhaus and Finger^15^.

### Determination of Drought sensitive Growth Stages

#### Plant Growth Stage Modelling (GDD)

**Figure 1:**
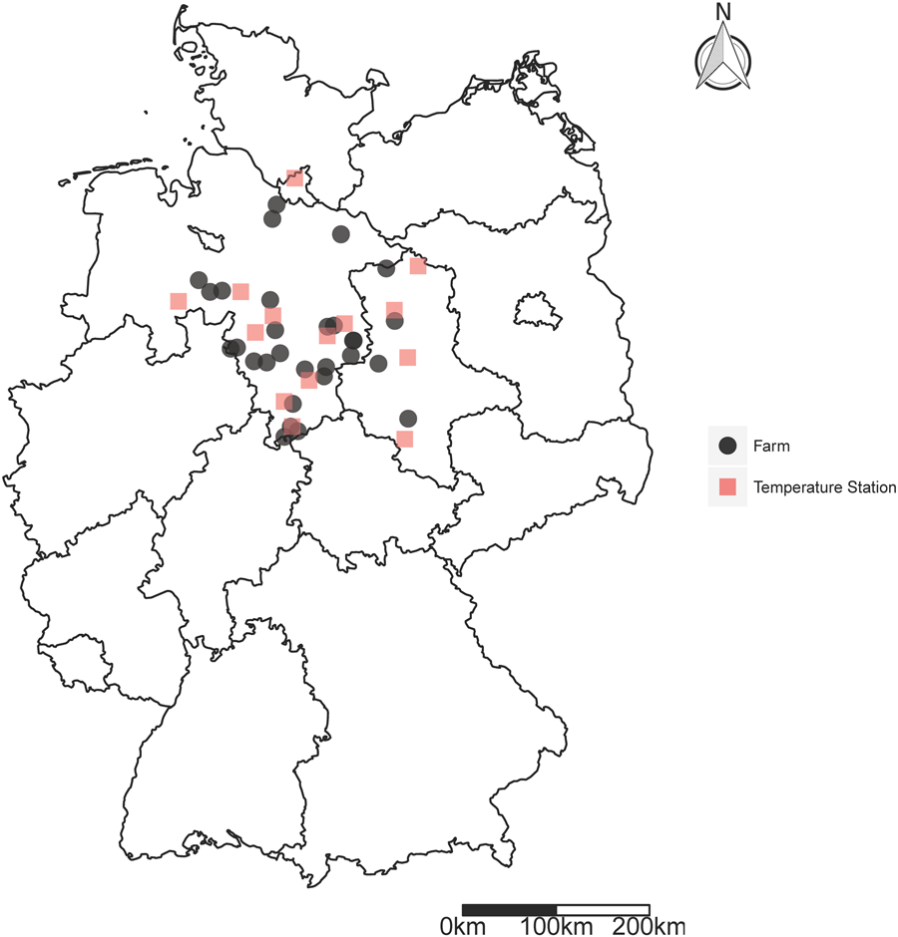
Location of Temperature measuring Weather Stations and Case Study Farms. The figure was created using the package ggplot2 (version 2.2.1.9)^62^ of the statistical software environment R-statistics (version 3.3.2).

First, we use a WII conditioned using plant growth stage modelling approach, i.e. growing degree days (GDD) as suggested by Conradt *et al*.^14^ (See Figure 1 for location information of temperature weather stations). The occurrence of different crop growth stages are calculated based on required air temperature loads (thermal time). This approach is denoted subsequently as ‘GDD’. Therefore, we take average seeding dates and calculate based on these all following growth stages.

5 $$GDD=\,\sum _{n=1}^{N}\,{\rm{\max }}(\min \,\{{H}_{n}^{av},{H}^{up}\}-{H}^{base},0)$$

We thus sum up mid-range daily air temperature H^av^ (${H}^{av}=\frac{{H}^{min}+{H}^{max}}{2}$; with ${H}^{min}\,{\rm{and}}\,{H}^{max}$ being the daily minimum and maximum air temperature respectively) if it is greater than H^base^ = 3 °C and lower than H^up^ = 22 °C. If H^av^ exceeds H^up^, we take H^up^ as GDD value, as growth is assumed to remain static then^28^. After reaching a GDD threshold, the plant is assumed to start a new growth stage. For our study region, we rely on literature values of these thresholds, see Table 4 for an overview. We consciously decided to rely on literature values only, to ensure a minimum of transaction costs and to propose an easy to implement, highly transparent and cheap insurance product.

**Table 4:** GDD Thresholds for different Growth Stages.

Phase	Assumption	Source
Seeding Date	15^th^ Oct	Chamber of Agriculture North Rhine-Westphalia^60^
Stem Elongation	659 °C	Miller *et al*.^61^
Anthesis	1,150 °C	Torriani *et al*.^25^

GDD values are used to identify the start of the ‘stem elongation’ growth stage and obtain the start date d_start;GDD_ for equation 5. We then repeat the procedure for the ‘anthesis’ growth period and get the end value d_end;GDD_ for equation 5. We decided to use this simple GDD model as this was applied in previous studies on WII^13,14^ and it is straightforward in implementation. From farmer’s perspective, the WII must be easy to understand and straight forward in its payout determination, a more complex growth stage model might counteract this requirement^16,17^.

### Yearly Phenology Reporter

**Figure 2:**
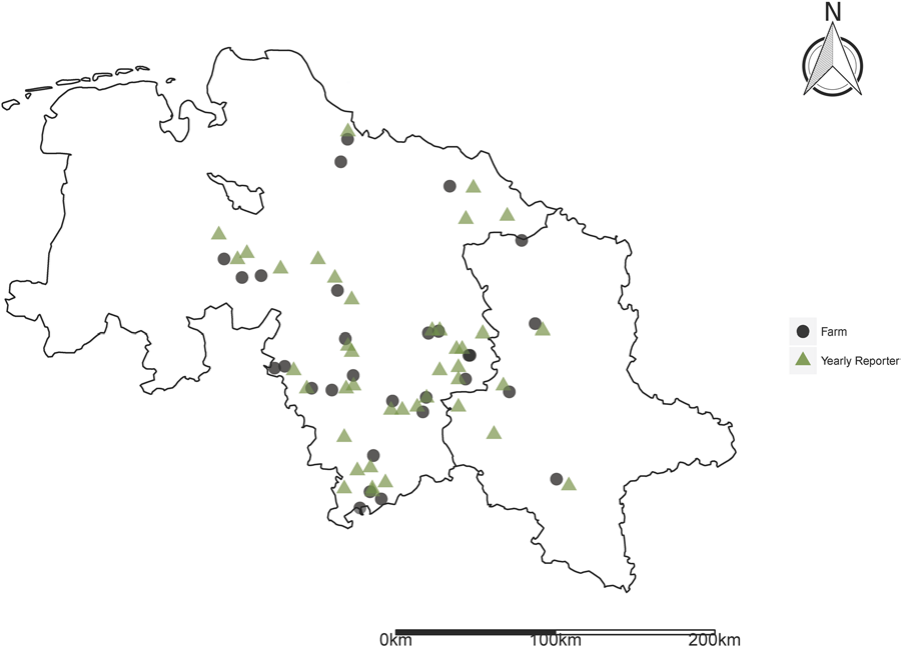
Location of Yearly Reporters. The figure was created using the package ggplot2 (version 2.2.1.9)^62^ of the statistical software environment R-statistics (version 3.3.2).

Second, we condition WII based on phenological observations that indicate growth stages in a particular region reported at the end of the year (see Fig. 2 for location information).

Deutscher Wetterdienst provides occurrence dates of growth stages for a variety of plants (ftp://ftp-cdc.dwd.de/). Within a basis network 1,200 reporters report on a yearly basis whereas 400 of these report their findings immediately. For wheat only, the former reporter dataset consist of ~650,000 observations. Taking into account that observations are available for over 20 different crops and immediate reporter data is published in real time, this phenology data constitutes a data source of large potential interest for various agricultural applications. Reports include growth information of wild growing but also agricultural flora cultivated under real-world (i.e. non-experimental) conditions. Observers check their reporting area two to three times a week and on a daily basis in rapid plant development periods. Untypical topographic points as well as unusual field conditions (climatic or cultivation anomalies) should be avoided. The data of single reporters is cross checked with surrounding reporters within the same natural region before publishing^47,48^ (within these natural regions plant growth conditions are similar). Similar public networks are available for various other major crop insurance markets (See van Vliet *et al*.^33^, for Europe, Morellato *et al*.^34^ for South and Central America and www.usapn.org for the US).

Our methodology here closely follows Dalhaus and Finger^15^. However, in contrast to this study, we focus solely and more detailed on one source of basis risk (temporal) and compare existing and propose new approaches coping with this issue. This data source provides high quality data, however with the drawback of being reported only at the end of the year. We use phenological observations of stem elongation and ear emergence to determine d_start; yea_ and d_end; yea_. As the growth stage of anthesis is not reported in the underlying data, we focus on the ear emergence growth stage that is closest to the anthesis stage.

The Yearly Reporters observe a reference field cultivated under practical conditions and capture a phenological phase, when about 50% of all plants reached it^47^. The findings are published online at the end of each year. Insurance payout can thus not be triggered directly after weather occurrence, but only when phenology reporters’ data is available.

### Immediate Phenology Reporter

**Figure 3:**
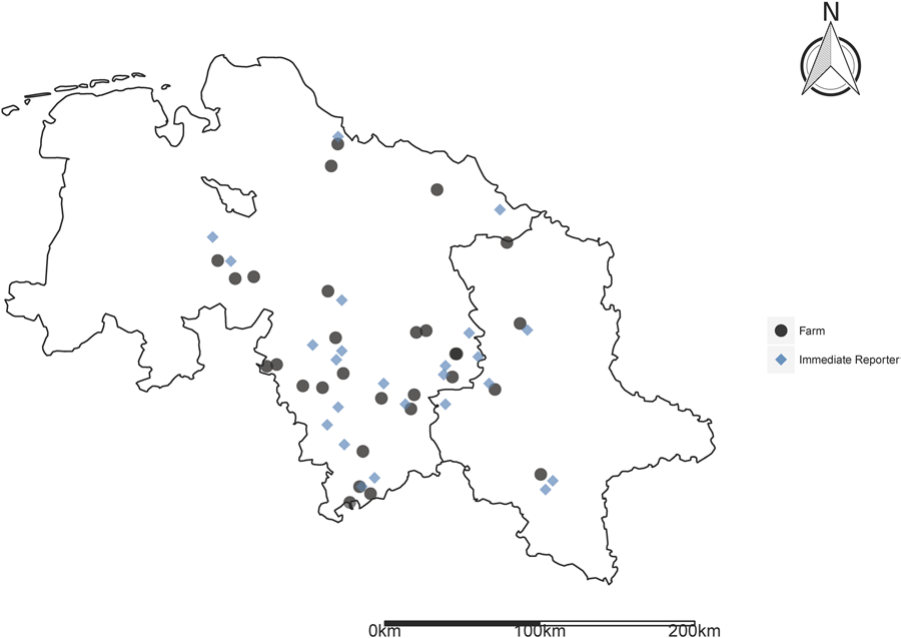
Location of Immediate Reporters. The figure was created using the package ggplot2 (version 2.2.1.9)^62^ of the statistical software environment R-statistics (version 3.3.2).

Third, we use an alternative source of phenological observations that comprises a live publishing reporting network (see Fig. 3 for location information). This third database would thus provide a substantially sooner payout in case of adverse weather events but comes with a considerably lower reporting density^48^. Comparing the Yearly and Immediate Reporter networks thus allows to reflect the balance between quality of the index (reporting density) and the timing of indemnification. Despite its potential, this study is the first considering this latter database in WII context.

The Immediate Reporters’ network of the Deutscher Wetterdienst contains around 400 reporters publishing phenological development right after the first occurrence of a growth stage. In contrast to Yearly Reporters, all sites within a radius of up to 5 km are considered, to give an impression of plant development in a wider reporting area. Immediate Reporters report the first occurrence of a growth stage within their reporting area. The Immediate Reporters’ network is especially implemented for the use in agricultural consultancy^47^.

Table 5 summarizes which growth stages are captured within the different reporting networks. Phases with high relevance for drought risks are III Stem Elongation -IV Ear Emergence for both reporting networks. This period captures drought risk during reproductive phases.

**Table 5:** Observed Phenological Phases of Immediate and Yearly Reporters.

No.	Yearly Reporters	Immediate Reporters
I	Tilling	Tilling
II	Seedling Growth	Seedling Growth
III	Stem elongation	Stem elongation
IV	Ear emergence	Ear emergence
V	Milk ripeness	
VI	Yellow ripeness	Yellow ripeness
VII	Harvest	Harvest

Source:^48^.

To precisely account for regional specifics, we use natural regions which were originally defined by Meynen and Schmitthüsen^49^ to find farm specific appropriate reporters. For the Immediate Reporters case we dropped four farms from the analysis for which no Immediate Reporters’ data was available within the natural region during the whole study period. For cases in which single year phenology reports were not available, an imputation strategy was applied where the mean value of occurrence dates across the available years was used as estimate.

### Performance testing

As risk management tool, a WII product is assumed to reduce farmers’ financial exposure to weather risk. In this context, the risk reducing properties of the insurance strongly depend on i) basis risk that affects insurance efficacy and ii) decision makers’ risk attitude that reflects farmers’ individual valuation of risk reducing properties of WII. To test the potential of different WII products to reduce temporal basis risk, we assess farmers’ expected utility of their crop production and implement different scenarios of risk aversion. Consequently, the insurance product providing highest expected utility is assumed to provide highest reduction of temporal basis risk. Within this framework, the utility function converts yearly monetary terminal wealth realizations into farm individual utility values, depending on level of risk aversion. Along these lines, we assume decreasing absolute risk aversion and use a power utility function to display farmers’ downside risk averse preferences (for recent examples in index insurance context see Dalhaus and Finger,^15^, Berg *et al*.^50^, and Leblois *et al*.^51^ and for a general motivation of the utility function Di Falco and Chavas^52,53^ and Finger^54^). To account for these differences we test several coefficients of relative risk aversion α ∈ [0, 0.5, 1, 2, 3, 4] ranging from risk neutral to extremely risk averse^55^. Assuming that farmers only hold the assets initial wealth ${W}_{0}$, wheat production and index insurance results in yearly terminal wealth ${W}_{tik}$:

6 $${W}_{tik}=P\cdot {y}_{ti}+{\pi }_{tik}^{put}-{{\rm{\Gamma }}}_{ik}+{W}_{0}$$

We used direct payments of 280 €/ha as initial wealth proxy. Hence the farm individual yearly utility is determined by:

7 $${{\rm{U}}}_{k{\rm{\alpha }}it}({W}_{tik})=\{\begin{array}{c}\frac{{W}_{tik}^{1-{\rm{\alpha }}}}{1-{\rm{\alpha }}}\,if\,\alpha \ne 1\\ \,\mathrm{ln}({W}_{tik})\,if\,\alpha =1\end{array}$$

This results in an utility value ${{\rm{U}}}_{k{\rm{\alpha }}it}$ for each WII product, year *t*, farm *i* and level of risk aversion α. The mean values across all years reflect the expected utility $E{{\rm{U}}}_{k{\rm{\alpha }}i}$ of farm *i*, insurance *k* and level of risk aversion α. Subsequently, we test insurance products against each other across different levels of risk aversion. More specifically, we use a non-parametric one sided paired Wilcoxon rank sum test to account for the ordinal nature of utility values^6,13^. More specifically, ordinal nature implies that expected utility values might only be compared with respect to their rank but not with respect to their absolute difference.

Assuming a fair premium, the insurance premium ${{\rm{\Gamma }}}_{ik}$ is equal to the expected payout. Burn rate pricing is used based on a bootstrapping procedure with 10,000 draws^56^. More specifically, we draw from the historical realizations of the insurance payouts during the period of study and take the average values of those draws. We moreover use a constant premium during the whole period of study as we do not expect changes in the risk exposure, e.g. due to climate change, to change our results. For implementing a marketable insurance product we refer to Kapphan *et al*.^13^, who include climate change scenarios in the pricing of WII.

### Weather Data

The underlying weather data was provided by the Deutscher Wetterdienst, an independent state institution. Hence data provision is transparent and comprehensible for policyholders (§ 1, Law of the Deutscher Wetterdienst). For index calculation two weather variables are necessary. First, precipitation data to determine daily rainfall. Second, air temperature data to find growth stages with GDD approach. For both variables we chose nearby weather stations with an average distance between farms and stations of 8.5 km for precipitation and 22.06 km for air temperature stations. All weather data was freely available under ftp://ftp-cdc.dwd.de/. Table 6 gives an overview of precipitation sums calculated using the different approaches of plant growth determination. The considerably higher mean precipitation during the GDD estimated growth phases is a result of the fact that this approach estimates stem elongation date systematically too early. All weather data and code used are available in the online supplementary information.

**Table 6:** Summary Statistics of Precipitation Sums in Growth Phases.

Growth Stage determination	Growth Stage	Mean	Standard deviation	Coefficient of Variation
GDD	Stem Elongation – Anthesis	108.77	53.40	0.49
Yearly Reporter	Stem Elongation – Ear Emergence	68.56	36.44	0.53
Immediate Reporter	Stem Elongation – Ear Emergence	68.51	31.88	0.46

Table A1 of the online supplementary file displays a comparison between rainfall determination approaches using Pearson correlation. While precipitation within reported phases of Yearly and Immediate Reporters is relatively closely related (0.58), GDD based precipitation sums are only weakly correlated with these two (0.24; 0.16).

### Farm Level Yield Data

Our case study was carried out using winter wheat yield data together with latitude and longitude coordinates of 29 northern German crop farms (see Fig. 1 for location information). To consider technical change during the study period from 1996 to 2010, yield data was detrended using linear trends. For summary statistics see Table 7. For a more detailed description of the study area see Dalhaus and Finger^15^. Pelka and Musshoff^57^ give a more detailed motivation of why linear detrending was used. They conclude from Heimfarth *et al*.^58^ that considering more robust regression approaches^59^ did not lead to differences in the results.

**Table 7:** Summary Statistics of Wheat Yields.

Summary statistics yield data		
Number of Farms		29
Minimum	[dt^a^/ha]	45.51
Maximum	[dt/ha]	132.00
Mean	[dt/ha]	86.91
Median	[dt/ha]	86.00
Standard deviation	[dt/ha]	14.47
Coefficient of Variation		0.17

Source: Dalhaus and Finger^15^.

^a^dt denotes deciton, i.e. 100 kg.

### Data Availability Statement

Attached to this paper we provide all data used and the underlying R-statistics code to fully replicate our results Execute for that the”Temporal Basis Risk.R” Code file which connects to the underlying datasets in “online Appendix.xlsx”.

## Electronic supplementary material

Supplementary Information

Temporal Basis Risk - R Code

online Appendix
