# Supplementary material for: Phenology Information Contributes to Reduce Temporal Basis Risk in Agricultural Weather Index Insurance
Source: Sci Rep. 2018 Jan 8;8:46. doi: 10.1038/s41598-017-18656-5 (PMC5758701; doi:10.1038/s41598-017-18656-5)
Supplement: Supplementary file 1 — Supplementary Information [file 41598_2017_18656_MOESM1_ESM.pdf]

Phenology Information Contributes to Reduce Temporal Basis Risk in Agricultural Weather Index Insurance

Tobias Dalhaus[1*], Oliver Musshoff[2], Robert Finger[1]

[1] Agricultural Economics and Policy Group, ETH Zürich, Sonneggstrasse 33, 8092 Zurich, Switzerland

[2] Department of Agricultural Economics and Rural Development, Georg August University Göttingen, Platz der Göttinger Sieben 5, 37073 Göttingen, Germany

* Correspondence to tdalhaus@ethz.ch

ONLINE SUPPLEMENTARY INFORMATION

Table A1: Correlation Matrix: Precipitation in growing phases

	GDD	Yearly Reporter	Immediate Reporter
GDD	1		
Yearly Reporter	0.24	1	
Immediate Reporter	0.16	0.58	1

Summary Results

To give a general overview about the differences across the growth stage estimation approaches, table A2 displays temporal gaps between the estimated timings. Hence, the GDD approach systematically estimates the occurrence of the 'stem elongation growth' stage ($d_{start;GDD}$) around a month earlier than the two reporting networks observe. In single outliers' cases these difference can increase up to 132 days, which leads to unrealistic dates. Note that all estimated (GDD) and observed (Yearly and Immediate Reporters network) dates are included in the online appendix.

For the second growing stage of interest, GDD estimates 'anthesis' ($d_{end;GDD}$) while the reporters only capture the actually earlier but less drought sensitive 'ear emergence' growing stage ($d_{end;imm}$ $d_{end;year}$). GDD estimate is still around 20 days earlier than the reporters. However, this misspecification is mainly caused by the former mentioned issue of estimating 'stem elongation' timing.

The median difference between Yearly and Immediate phenology Reporters is close to zero days for both phases. However, for single reports this difference can be up to 68 days. These differences arise from distance between single yearly and immediate reporters' locations as well as different reporting strategies (see section 3.2).

Table A2: Differences in estimated timings in days

Approaches		Stem Elongation	Ear Emergence/ Anthesis
Difference between Yearly Reporters' and GDD Dates			
	Median	37	21
	Min	-6	-23
	Max	132	76
Difference between Immediate Reporters' and GDD Dates			
	Median	33	20
	Min	-26	-19
	Max	123	64
Difference between Yearly and Immediate Reporters' Dates			
	Median	2	0
	Min	-68	-26
	max	54	34

Table A3 summarizes WII contract parameters across the different approaches of growth stage determination. The aforementioned early estimated dates of the GDD approach lead to the fact that the insured rainfall period is longer and shifted into a period in which higher rainfall is more likely. Resulting strike levels that have to be undercut to trigger an insurance payout are higher compared to case when insuring via phenology reporting networks. Medium as well as maximum WII premium rates reflect a high drought risk exposure, as expected in this region. On average, an indemnification of the WII (i.e. net payouts are positive) in 4.10 out 15 years for the GDD approach, 6.03 years for Yearly and 5.28 years for Immediate Reports. Thus, farmers using WII based on phenology reporters' data receive an indemnification of 29% to 47% more likely compared to GDD case. Even though this might increase transaction costs, not too rare indemnification is seen as important determinant of the success of WII [1].

The differences in the variable 'number of insured farms' are due to the fact that we restricted the insurance only to be concluded if the sign of the estimated relationship between yield and rainfall was positive. That means, if our regression detected a higher negative influence of excessive rainfall compared to low rainfalls' influence in the given growing stage, we dropped these cases as we assumed the farmers not to conclude an insurance contract then. Furthermore, for 8 farms there was no Immediate Reporters' data available in the farms natural region.

Table A3: Summary Statistics of Insurance Contract Parameters for α=1 across all 29 case Study Farms.

Data Source	GDD	Yearly Reporter	Immediate Reporter
Strike Level [mm precipitation/m²]			
Median	191.64	122.91	125.31
Min	128.04	47.20	66.82
Max	1903.23	507.02	360.78
Premium [€/ha]			
Median	61.17	62.74	77.39
Min	7.78	8.89	16.71
Max	162.82	166.64	166.80
Average Number of positive net Payouts (payout minus premium; out of 15 years)			
Mean	4.10	6.03	5.28
Number of insured out of 29 farms*			
	15	24	21

*Note that we assumed the insurance contract to be concluded only if the slope coefficient of QR was positive and if phenology reporters' data was available.

For full information about the variables see the online appendix.

Table A4: Average Changes of Risk Premium in Percentage Terms, WII compared to uninsured Scenario

Coefficient of relative risk aversion r_r	Yearly Reporters vs. Uninsured	Immediate Reporters vs. Uninsured	GDD vs. Uninsured
0 (risk neutral)			
0.5	- 6.25	-2.19	- 0.34
1	- 6.39	-2.31	- 0.45
2	- 6.66	-2.52	- 0.68
3	- 6.91	- 2.72	- 0.91
4 (extremely risk averse)	- 7.14	- 2.89	- 1.13

Sensitivity Analyses

Table A5 Results RQ1: Tests for risk reducing properties of different WII compared to no insurance (Wheat Price changed to 20€/dt)

Coefficient of relative risk aversion r_r	H_0:$EU_{year} \geq EU_{noins}$	H_0:$EU_{imm} \geq EU_{noins}$	H_0:$EU_{GDD} \geq EU_{noins}$
	p- value		
0 (risk neutral)	0.62	0.29	0.93
0.5	$3.27 \cdot 10^{-2}$	$4.76 \cdot 10^{-2}$	0.85
1	$2.35 \cdot 10^{-2}$	$7.49 \cdot 10^{-3}$	0.60
2	$6.73 \cdot 10^{-3}$	$9.94 \cdot 10^{-3}$	0.60
3	$5.73 \cdot 10^{-3}$	$1.43 \cdot 10^{-2}$	0.56
4 (extremely risk averse)	$4.11 \cdot 10^{-3}$	$1.31 \cdot 10^{-2}$	0.49

Table A6 Results RQ2: Comparing risk reducing properties between WII (Wheat Price changed to 20€/dt)

Coefficient of relative risk aversion r_r	H_0:$EU_{year} \geq EU_{imm}$	H_0:$EU_{year} \geq EU_{GDD}$	H_0:$EU_{imm} \geq EU_{GDD}$
	p-value		
0 (risk neutral)	0.50	0.24	0.17
0.5	0.27	$3.77 \cdot 10^{-2}$	$6.32 \cdot 10^{-2}$
1	0.33	$3.77 \cdot 10^{-2}$	$4.46 \cdot 10^{-2}$
2	0.12	$2.24 \cdot 10^{-2}$	$5.02 \cdot 10^{-2}$
3	0.11	$2.11 \cdot 10^{-2}$	$6.32 \cdot 10^{-2}$
4 (extremely risk averse)	0.10	$1.98 \cdot 10^{-2}$	$9.20 \cdot 10^{-2}$

Table A7 Results RQ1: Tests for risk reducing properties of different WII compared to no insurance (Wheat Price changed to 10€/dt)

Coefficient of relative risk aversion r_r	H_0:$EU_{year} \geq EU_{noins}$	H_0:$EU_{imm} \geq EU_{noins}$	H_0:$EU_{GDD} \geq EU_{noins}$
	p- value		
0 (risk neutral)	0.62	0.29	0.93
0.5	$4.46 \cdot 10^{-2}$	$8.22 \cdot 10^{-2}$	0.88
1	$3.06 \cdot 10^{-2}$	$9.05 \cdot 10^{-3}$	0.69
2	$7.88 \cdot 10^{-3}$	$9.94 \cdot 10^{-3}$	0.62
3	$6.21 \cdot 10^{-3}$	$1.31 \cdot 10^{-2}$	0.53
4 (extremely risk averse)	$4.88 \cdot 10^{-3}$	$1.31 \cdot 10^{-2}$	0.58

Table A8 Results RQ2: Comparing risk reducing properties between WII (Wheat Price changed to 10€/dt)

Coefficient of relative risk aversion r_r	H_0:$EU_{year} \geq EU_{imm}$	H_0:$EU_{year} \geq EU_{GDD}$	H_0:$EU_{imm} \geq EU_{GDD}$
		p-value	
0 (risk neutral)	0.50	0.24	0.17
0.5	0.30	$3.37 \cdot 10^{-2}$	$5.97 \cdot 10^{-2}$
1	0.35	$3.98 \cdot 10^{-2}$	$3.94 \cdot 10^{-2}$
2	0.16	$2.53 \cdot 10^{-2}$	$4.19 \cdot 10^{-2}$
3	0.11	$2.11 \cdot 10^{-2}$	$5.97 \cdot 10^{-2}$
4 (extremely risk averse)	0.11	$1.86 \cdot 10^{-2}$	$7.06 \cdot 10^{-2}$

Table A9 Results RQ1: Tests for risk reducing properties of different WII compared to no insurance (initial wealth changed to 200€/ha)

Coefficient of relative risk aversion r_r	H_0:$EU_{year} \geq EU_{noins}$	H_0:$EU_{imm} \geq EU_{noins}$	H_0:$EU_{GDD} \geq EU_{noins}$
	p- value		
0 (risk neutral)	0.62	0.29	0.93
0.5	$3.27 \cdot 10^{-2}$	$4.42 \cdot 10^{-2}$	0.84
1	$2.35 \cdot 10^{-2}$	$8.24 \cdot 10^{-3}$	0.60
2	$6.73 \cdot 10^{-3}$	$9.94 \cdot 10^{-3}$	0.58
3	$5.73 \cdot 10^{-3}$	$1.43 \cdot 10^{-2}$	0.56
4 (extremely risk averse)	$3.78 \cdot 10^{-3}$	$1.31 \cdot 10^{-2}$	0.49

Table A10 Results RQ2: Comparing risk reducing properties between WII (initial wealth changed to 200€/dt)

Coefficient of relative risk aversion r_r	H_0:$EU_{year} \geq EU_{imm}$	H_0:$EU_{year} \geq EU_{GDD}$	H_0:$EU_{imm} \geq EU_{GDD}$
	p-value		
0 (risk neutral)	0.50	0.24	0.17
0.5	0.27	$3.77 \cdot 10^{-2}$	$6.32 \cdot 10^{-2}$
1	0.32	$3.77 \cdot 10^{-2}$	$4.73 \cdot 10^{-2}$
2	0.12	$2.24 \cdot 10^{-2}$	$5.02 \cdot 10^{-2}$
3	0.11	$2.11 \cdot 10^{-2}$	$6.32 \cdot 10^{-2}$
4 (extremely risk averse)	0.10	$1.86 \cdot 10^{-2}$	$8.74 \cdot 10^{-2}$

Table A11 Results RQ1: Tests for risk reducing properties of different WII compared to no insurance (initial wealth changed to 350€/ha)

Coefficient of relative risk aversion r_r	H_0:$EU_{year} \geq EU_{noins}$	H_0:$EU_{imm} \geq EU_{noins}$	H_0:$EU_{GDD} \geq EU_{noins}$
	p- value		
0 (risk neutral)	0.62	0.29	0.93
0.5	$3.48 \cdot 10^{-2}$	$7.22 \cdot 10^{-2}$	0.84
1	$2.87 \cdot 10^{-2}$	$9.05 \cdot 10^{-3}$	0.64
2	$6.73 \cdot 10^{-3}$	$1.09 \cdot 10^{-2}$	0.60
3	$5.73 \cdot 10^{-3}$	$1.43 \cdot 10^{-2}$	0.51
4 (extremely risk averse)	$4.86 \cdot 10^{-3}$	$1.31 \cdot 10^{-2}$	0.56

Table A12 Results RQ2: Comparing risk reducing properties between WII (initial wealth changed to 350€/dt)

Coefficient of relative risk aversion r_r	H_0:$EU_{year} \geq EU_{imm}$	H_0:$EU_{year} \geq EU_{GDD}$	H_0:$EU_{imm} \geq EU_{GDD}$
	p-value		
0 (risk neutral)	0.50	0.24	0.17
0.5	0.27	$3.37 \cdot 10^{-2}$	$6.32 \cdot 10^{-2}$
1	0.36	$3.77 \cdot 10^{-2}$	$3.71 \cdot 10^{-2}$
2	0.15	$2.68 \cdot 10^{-2}$	$4.46 \cdot 10^{-2}$
3	0.10	$2.11 \cdot 10^{-2}$	$5.97 \cdot 10^{-2}$
4 (extremely risk averse)	0.11	$1.86 \cdot 10^{-2}$	$7.86 \cdot 10^{-2}$

Table A13 Results RQ1: Tests for risk reducing properties of different WII compared to no insurance (loading 10%)

Coefficient of relative risk aversion r_r	H_0:$EU_{year} \geq EU_{noins}$	H_0:$EU_{imm} \geq EU_{noins}$	H_0:$EU_{GDD} \geq EU_{noins}$
	p- value		
0 (risk neutral)	0.99	0.99	1.00
0.5	0.99	0.99	1.00
1	0.99	0.99	1.00
2	0.99	0.99	1.00
3	0.99	0.99	1.00
4 (extremely risk averse)	0.95	0.99	0.99

When adding a loading factor of 10% to the insurance premiums, none of the insurances reduces the financial exposure to risk any longer. This was to be expected, as we only insure a single peril. For a marketable insurance product multiple weather risks should be combined and a whole farm risk management strategy should be developed. As we only compare the different options GDD, yearly reporter and immediate reporter to reduce temporal basis risk of the rainfall component of a WII, results displayed in table A13 do not change the general conclusions drawn in the main paper.

Table A14 Results RQ2: Comparing risk reducing properties between WII (loading 10%)

Coefficient of relative risk aversion r_r	H_0:$EU_{year} \geq EU_{imm}$	H_0:$EU_{year} \geq EU_{GDD}$	H_0:$EU_{imm} \geq EU_{GDD}$
	p-value		
0 (risk neutral)	0.92	0.53	0.19
0.5	0.88	0.36	0.17
1	0.81	0.23	0.13
2	0.69	$6.03 \cdot 10^{-2}$	0.10
3	0.46	$4.16 \cdot 10^{-2}$	$7.57 \cdot 10^{-2}$
4 (extremely risk averse)	0.34	$3.19 \cdot 10^{-2}$	0.11

When adding a loading factor of 10% to the insurance premium, the superiority of yearly and immediate reporter based WII compared to GDD vanishes. This is due to the fact, that the GDD based insurance comes with a lower overall absolute premium compared to yearly and immediate reporter based WII as shown in table A3. Hence, the loading factor is more pronounced in the reporter based WIIs as the absolute loading is higher in these cases. The resulting loss in the expected value of revenues is thus higher in the case of yearly and immediate reporter based WIIs, when adding a loading factor. This higher loss in revenues drives the expected utility calculations for table A14. Resulting, the three insurances are no longer comparable when adding a loading factor. Tables A15 and A16 show the results when adding an absolute loading of 10€/ha instead of a loading factor, coming to similar results as displayed in the main paper.

Table A15 Results RQ1: Tests for risk reducing properties of different WII compared to no insurance (loading 10€/ha)

Coefficient of relative risk aversion r_r	$H_0{:}EU_{year} \geq EU_{noins}$	$H_0{:}EU_{imm} \geq EU_{noins}$	$H_0{:}EU_{GDD} \geq EU_{noins}$
	p- value		
0 (risk neutral)	0.99	0.99	1.00
0.5	0.99	0.99	1.00
1	0.99	0.99	1.00
2	0.99	0.99	1.00
3	0.99	0.99	1.00
4 (extremely risk averse)	0.99	0.99	0.99

Table A16 Results RQ2: Comparing risk reducing properties between WII (loading 10€/ha)

Coefficient of relative risk aversion r_r	H_0:$EU_{year} \geq EU_{imm}$	H_0:$EU_{year} \geq EU_{GDD}$	H_0:$EU_{imm} \geq EU_{GDD}$
	p-value		
0 (risk neutral)	0.87	0.36	0.05
0.5	0.83	$1.31 \cdot 10^{-3}$	$1.68 \cdot 10^{-3}$
1	0.83	$5.80 \cdot 10^{-4}$	$3.05 \cdot 10^{-4}$
2	0.60	$4.27 \cdot 10^{-4}$	$2.14 \cdot 10^{-4}$
3	0.50	$3.05 \cdot 10^{-4}$	$4.27 \cdot 10^{-4}$
4 (extremely risk averse)	0.45	$3.05 \cdot 10^{-4}$	$3.05 \cdot 10^{-4}$

References

Vargas-Hill, R., Robles, M. Ceballos, F. Demand for a Simple Weather Insurance Product in India: Theory and Evidence. *Am. J. Agr. Econ*. **98** (4), 1250-1270 (2016).
